# Retrospective quality metrics review of stereotactic radiosurgery plans treating multiple targets using single‐isocenter volumetric modulated arc therapy

**DOI:** 10.1002/acm2.12869

**Published:** 2020-04-02

**Authors:** Yunfeng Cui, Hao Gao, Jiahan Zhang, John P. Kirkpatrick, Fang‐Fang Yin

**Affiliations:** ^1^ Department of Radiation Oncology Duke University Durham NC USA; ^2^ Department of Radiation Oncology Emory University Atlanta GA USA

**Keywords:** normal brain V12Gy, plan quality, single‐isocenter multi‐target VMAT

## Abstract

**Purpose:**

To characterize key plan quality metrics in multi‐target stereotactic radiosurgery (SRS) plans treated using single‐isocenter volumetric modulated arc therapy (VMAT) in comparison to dynamic conformal arc (DCA) plans treating single target. To investigate the feasibility of quality improvement in VMAT planning based on previous planning knowledge.

**Materials and methods:**

97 VMAT plans of multi‐target and 156 DCA plans of single‐target treated in 2017 at a single institution were reviewed. A total of 605 targets were treated with these SRS plans. The prescription dose was normalized to 20 Gy in all plans for this analysis. Two plan quality metrics, target conformity index (CI) and normal tissue volume receiving more than 12 Gy (V12Gy), were calculated for each target. The distribution of V12Gy per target was plotted as a function of the target volume. For multi‐target VMAT plans, the number of targets being treated in the same plan and the distance between targets were calculated to evaluate their impact on V12Gy. VMAT plans that had a large deviation of V12Gy from the average level were re‐optimized to determine the possibility of reducing the variation of V12Gy in VMAT planning.

**Results:**

Conformity index of multi‐target VMAT plans were lower than that of DCA plans while the mean values of 12 Gy were comparable. The V12Gy for a target in VMAT plan did not show apparent dependence on the total number of targets or the distance between targets. The distribution of V12Gy exhibited a larger variation in VMAT plans compared to DCA plans. Re‐optimization of outlier plans reduced V12 Gy by 33.9% and resulted in the V12Gy distribution in VMAT plans more closely resembling that of DCA plans.

**Conclusion:**

The benchmark data on key plan quality metrics were established for single‐isocenter multi‐target SRS planning. It is feasible to use this knowledge to guide VMAT planning and reduce high V12Gy outliers.

## 1 INTRODUCTION

Brain metastases occur in many cancer patients with different incident rates depending on cancer type.^1^, ^2^ Patients who develop brain metastases are associated with shorter life expectancy.^3^ Surgery, whole‐brain radiation therapy (WBRT), and stereotactic radiosurgery (SRS) are the main approaches to manage patients with brain metastases. Stereotactic radiosurgery is preferred over WBRT for the patients with a limited number (1–4) of brain metastases^4^, ^5^, ^6^ due to the fact that WBRT can cause neurocognitive toxicity and offers no overall survival advantage when compared to SRS. With technology development in recent years, treating more brain metastases with SRS became feasible and more patients who would have been treated with WBRT historically are now routinely being treated with SRS alone.^7^, ^8^

Single‐isocenter volumetric modulated arc therapy (VMAT) technique treating multiple lesions at the same time made the multi‐target SRS more practical by dramatically reducing the treatment time compared to treating each target separately.^9^, ^10^, ^11^, ^12^, ^13^ With the ability of beam modulation and inverse optimization, the single‐isocenter multi‐target (SIMT) VMAT plan can achieve good dose conformity to targets compared with other SRS treatment techniques.^14^, ^15^ On the other hand, because of the complexity of VMAT planning the plan quality metrics across different planning systems and planners could be less consistent.^16^, ^17^, ^18^ There is a concern especially for the dose fall‐off gradient and low dose spread in SIMT VMAT plan due to the potential large opening of multi‐leaf collimator (MLC) causing irradiation of normal tissue in between targets.^19^, ^20^, ^21^ Among those dosimetric metrics, normal brain volume receiving more than 12 Gy (V12Gy) is a biomarker that has been identified to correlate with normal tissue necrosis.^7^, ^22^, ^23^ A benchmarking data on SRS plan dosimetric metrics will provide the planners guidance about what can be achieved for specific cases.^24^, ^25^, ^26^

The purpose of this study is to retrospectively characterize CI and V12Gy in multi‐target VMAT plans treated in the past, and review them in comparison to metrics from single‐target dynamic conformal arc (DCA) plans for any indication. The study aimed to establish an average performance level of these key plan quality metrics and investigate how they are impacted by the number of total targets in the same plan or the distance between targets. Based on the established planning knowledge, the study also explored the feasibility of improving VMAT plan quality when a plan does not meet the average performance level.

## 2 MATERIALS AND METHODS

### 2.A Data

Two hundred and fifty‐three linear accelerator‐based single‐fraction SRS plans treated in 2017 at a single institution were reviewed. A total of 605 lesions were included in these plans as treatment targets. One hundred and fifty‐six plans were DCA technique treating single target in each plan, and 97 plans used a VMAT technique to treat multiple targets simultaneously in a single plan. All DCA plans were generated in iPlan treatment planning system (version 4.5.5, Brainlab, Feldkirchen, Germany) and all VMAT plans were generated in Eclipse treatment planning system (version 13.6, Varian Medical Systems, Palo Alto, CA) for linear accelerators equipped with Varian HD120 MLC (Varian Medical Systems, Palo Alto, CA). Dose calculation grid size was set to 1mm in both planning systems.

### 2.B Plan quality metrics

The prescription dose was normalized to 20Gy for all plans for this analysis. This means the prescription isodose line (100% isodose line) was set to 20Gy for each target for comparison. Two plan quality metrics, tumor conformity index (CI) and normal brain V12Gy, were calculated for each target. The CI is defined [Eq. (1)] as the ratio of prescription isodose volume (*V*_100%_) over the volume (*V*_PTV_) of planning target volume (PTV). Normal brain V12Gy is defined as 12 Gy isodose volume (60% isodose volume) subtracted by the volume of PTV. In multi‐target VMAT, CI and V12Gy were calculated for each target separately. In case that two isodose volumes from two closely located targets are connected, voxels in the connected isodose volume are assigned to a particular target based on the distance from the voxel to the surface of the target. Each voxel in the connected isodose volume is only assigned to one closest target, dividing the connected volume into two sub‐volumes. CI or V12Gy of each target is then calculated with the corresponding sub‐volume. Other metrics were also extracted in this study for supplemental information on plan quality evaluation. These metrics included target coverage, target max dose, and paddick conformity index (CI_Paddick_).^27^ CI_Paddick_ is defined by [Eq. (2)], where PTV_100%_ represents the volume of PTV covered by the prescription isodose.

(1) $$\text{CI}=\frac{V_{100\%}}{V_{\text{PTV}}}$$

(2) $${\text{CI}}_{\text{Paddick}}=\frac{{\text{PTV}}_{100\%}^{2}}{V_{\text{PTV}}\times V_{100\%}}$$

### 2.C Comparison between DCA and VMAT plans

The average and the standard deviation of CI and V12Gy were calculated for the targets in DCA plans and VMAT plans separately for comparison. Since the CI and V12Gy are target‐volume dependent, the targets were binned into a number of groups based on the volume and the statistics was acquired for each group. The Mann‐Whitney test (MATLAB, The MathWorks, Inc., Natick, MA) was used to calculate the significance of differences between DCA and VMAT plan populations.

The distribution of normal brain V12Gy as a function of target volume was plotted and the variation in V12Gy was compared between DCA and VMAT plans. For each target in VMAT plans, the surface distance of that target to the nearest other target and the number of targets being treated in the same plan were calculated to evaluate their impact on V12Gy.

### 2.D Re‐planning of outlier cases

Targets in VMAT plans that had a large deviation of V12Gy from average level were identified and the plans for these targets were re‐optimized to determine the possibility of reducing the variation of V12Gy in VMAT planning. The outlier cases were identified visually from the plot of V12Gy distribution as a function of the target volume. The re‐optimization of the plan kept the exact same beam arrangement as in the original plan (same energy, arc path, collimator angle, field size) but adjusted weighting in normal tissue constraints in the optimization process, knowing that better V12Gy should be achievable in these outlier cases. Key plan quality metrics (CI, target max dose, target coverage, and V12Gy) in re‐optimized plans were compared with those in original plans. The Wilcoxon matched‐pairs test (MATLAB, The MathWorks, Inc., Natick, MA) was used to calculate the significance of V12Gy improvement in the re‐optimized plans.

## 3 RESULTS

Mean value and standard deviation (SD) of CI and V12Gy for DCA plans and VMAT plans were listed in Table 1. The lesions were binned into different groups based on the size, and mean and SD values were calculated for each group. The *p* values resulting from Mann‐Whitney tests comparing DCA and VMAT groups were also included in the table. The VMAT plans consistently produced lower CI and kept the normal brain V12Gy per target comparable to DCA plans. Table 2 includes other dose‐volume metrics (target coverage, max dose, and CI_Paddick_) that are relevant to SRS planning.

**Table 1:** Mean and Standard Deviation of CI and V12Gy.

Target volume	CI (mean ± SD)		*P* value	V12Gy (cc) (mean ± SD)		*P* value
	DCA	VMAT		DCA	VMAT	
<0.2 cc	2.1 ± 0.3	1.8 ± 0.4	<0.001	1.2 ± 0.3	1.3 ± 0.5	0.183
0.2 cc–0.5 cc	1.9 ± 0.2	1.5 ± 0.2	<0.001	1.9 ± 0.4	2.2 ± 0.7	0.132
0.5 cc–1 cc	1.8 ± 0.3	1.4 ± 0.2	<0.001	3.5 ± 0.8	3.2 ± 0.7	0.089
1 cc–2 cc	1.6 ± 0.2	1.3 ± 0.2	<0.001	5.2 ± 1.0	4.7 ± 1.3	0.016
2 cc–4 cc	1.6 ± 0.1	1.3 ± 0.1	<0.001	7.4 ± 1.0	7.8 ± 1.8	0.225

CI, conformity index; V12Gy, normal brain volume receiving more than 12Gy; DCA, dynamic conformal arc; VMAT, volumetric modulated arc therapy; SD, Standard Deviation; *p* value, *p* value of Mann‐Whitney test between DCA and VMAT groups.

**Table 2:** Target coverage, max dose, and CI_Paddick_ for the targets included in this study.

	Target coverage (mean ± SD)	Max dose (mean ± SD)	CI_Paddick_ (mean ± SD)
DCA plans	99.9% ± 0.2%	111.4% ± 3.4%	0.56 ± 0.09
VMAT plans	99.7% ± 0.5%	114.8% ± 5.5%	0.65 ± 0.12
Overall	99.8% ± 0.4%	113.9% ± 5.3%	0.63 ± 0.12

Target coverage = Percentage of target volume that is covered by the prescription isodose; Max dose = Percentage of max dose inside target relative to the prescription dose; DCA = dynamic conformal arc; VMAT = volumetric modulated arc therapy; SD = Standard Deviation; CI_Paddick_ = paddick conformity index.

**Fig. 1:**
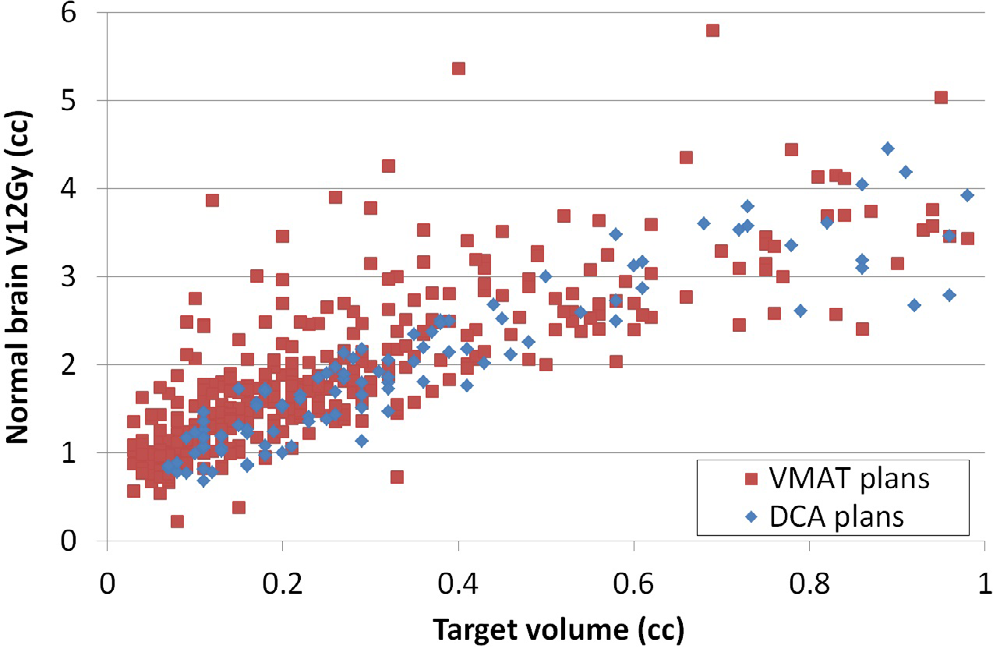
Distribution of normal brain V12Gy per target as a function of target volume for multi‐target VMAT plans and single‐target DCA plans. Red square represents the targets in VMAT plans, and blue diamond represents the targets in DCA plans. Targets with volume up to 1cc are included in this graph. DCA, dynamic conformal arc; VMAT, volumetric modulated arc therapy.

Figure 1 shows the distribution of normal brain V12Gy per target as a function of target volume for multi‐target VMAT plans and single‐target DCA plans. Targets with volume up to 1cc are included in this graph. In this figure, the VMAT plans showed more variation in V12Gy compared to DCA plans. There were a number of points in VMAT plans that deviated from the average level of V12Gy.

**Fig. 2:**
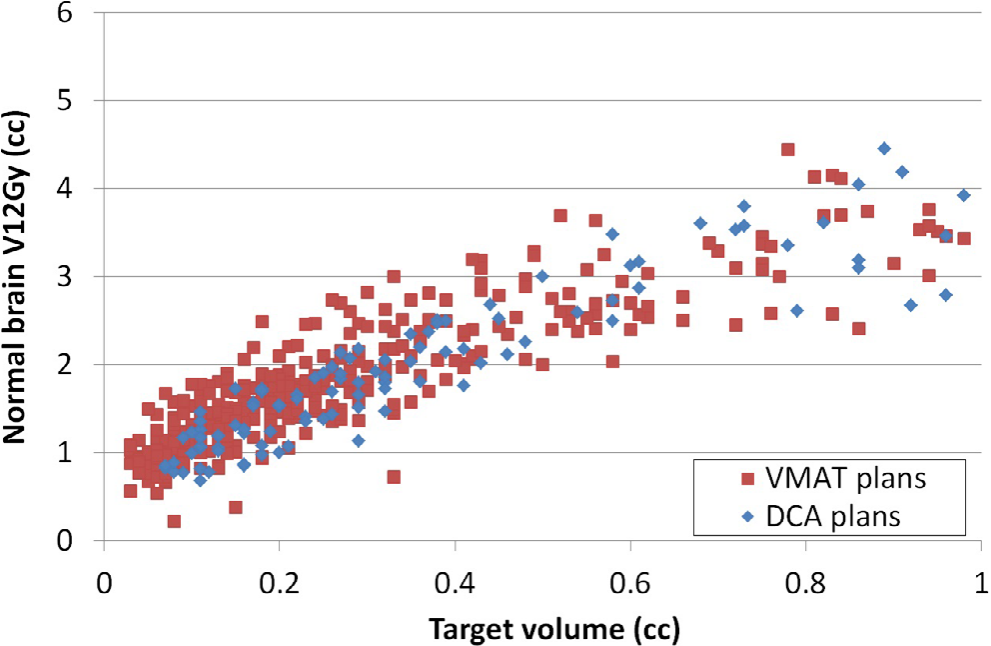
Distribution of normal brain V12Gy per target as a function of target volume after re‐planning of outlier cases. Red square represents the targets in VMAT plans, and blue diamond represents the targets in DCA plans. Targets with volume up to 1cc are included in this graph. DCA, dynamic conformal arc; VMAT, volumetric modulated arc therapy.

As described in the Method section, those outlier cases that had a large deviation from average V12Gy level in Fig. 1 were identified and the VMAT plans containing those targets were re‐optimized knowing that a better V12Gy should be achievable in those outlier cases. Figure 2 shows the distribution of normal brain V12Gy per target after the re‐optimization. Forty‐three targets in VMAT plans were selected for re‐planning based on Fig. 1, which resulted in the re‐optimization of eight plans that had 2, 4, 5, 7, 8, 9, 12, and 15 lesions, respectively. By comparing Figs. 1 and 2, it can be seen that the variation of V12Gy in VMAT plans was reduced after re‐planning and V12Gy distribution in VMAT plans more closely resembled that of DCA plans.

**Fig. 3:**
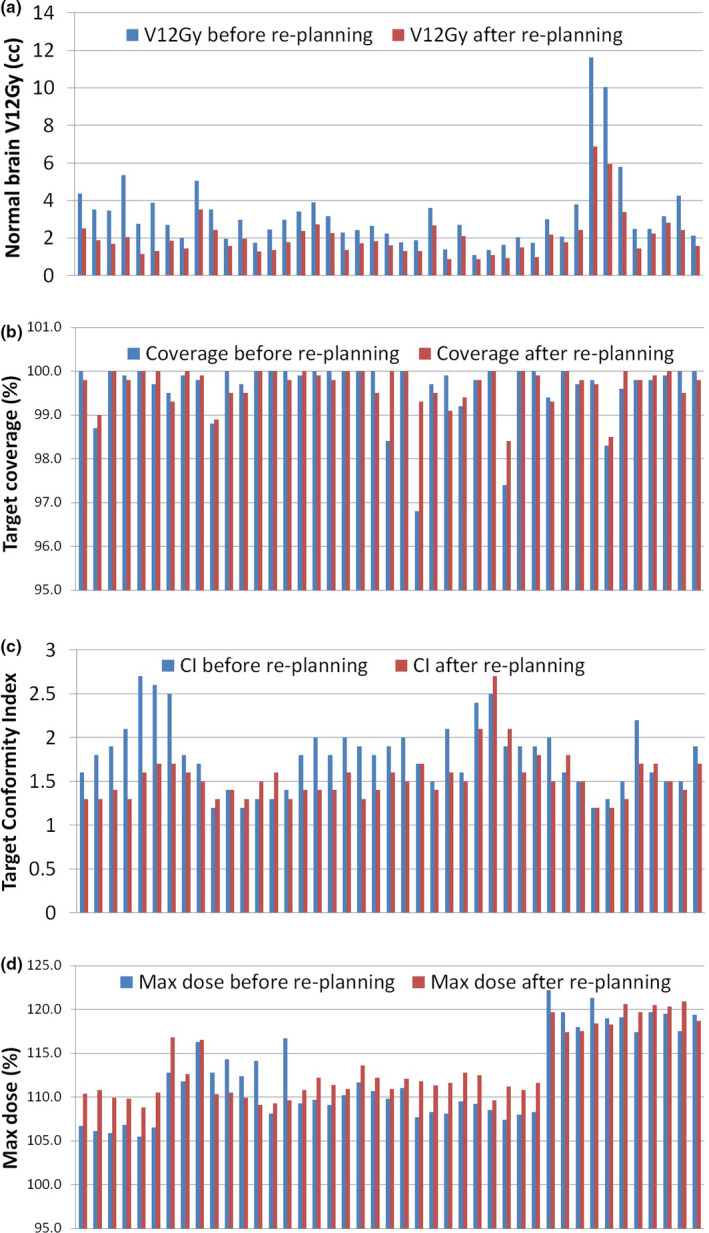
Comparison of dosimetric metrics for re‐planned cases before and after re‐planning. (a) normal brain V12Gy; (b) percentage volume of target covered by prescription dose; (c) Target Conformity Index (CI); (d) Target max dose.

Figure 3 shows a comparison of dosimetric metrics for re‐planned cases before and after re‐planning. The mean reduction of V12Gy in the re‐planned targets was 33.9% (range 10.5–66.1%), while other dosimetric metrics including CI, target coverage, and max dose were kept at the same level. The changes of V12Gy before and after re‐planning was statistically significant (*P* < 0.001, Wilcoxon matched pairs test).

**Fig. 4:**
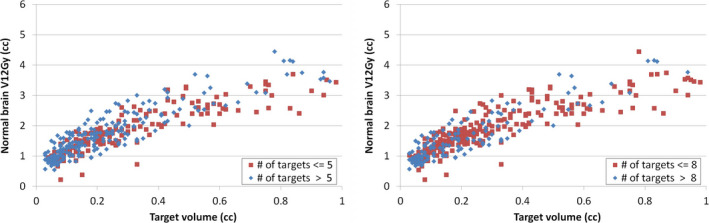
Normal brain V12Gy per target vs. number of targets being treated in the same plan. In the left image, red squares are the targets from plans that have 5 or less targets in same plan, and blue diamonds are the targets from plans that treated more than 5 targets at the same time. In right image, the threshold is 8.

Figure 4 shows the impact of number of simultaneously treated targets on the V12Gy of each target. The targets were separated into two groups based on the number of targets being treated in the same plan. The plots in Fig. 4 used cutoff numbers of 5 and 8. We didn't see apparent separation in between two groups in terms of normal brain V12Gy. We tested this cutoff number from 3 to 12 and got the same conclusion.

**Fig. 5:**
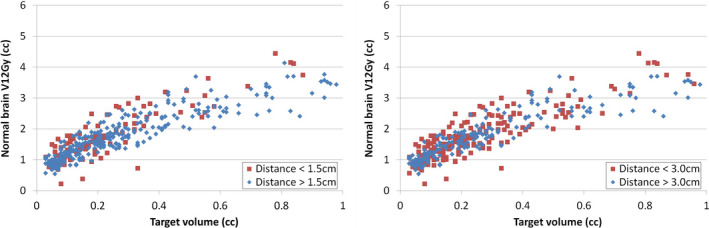
Normal brain V12Gy per target vs. surface distance of the target to the nearest other target. In the left image, red squares are the targets with a nearest surface distance less than 1.5cm, and blue diamonds are the targets with a nearest surface distance greater than 1.5cm. In the right image, the threshold is 3.0 cm.

Figure 5 shows the impact of surface distance of a target to its nearest other target on the V12Gy. The targets were separated into two groups based on the surface distance of the target to its nearest other target. The plots in Fig. 5 used cutoff values of 1.5 cm and 3.0 cm. There was no apparent separation in between two groups as shown in this figure and also when tested with different thresholds down to 0.5 cm.

## 4 DISCUSSION

This study was conducted in a retrospective manner by reviewing previous clinical plans to characterize important plan quality metrics and factors that impact these metrics. Clinical plans were carried out using either DCA or VMAT technique. Compared to DCA technique, there are much more factors that control a VMAT plan quality, which raised a clinical question if VMAT plans achieved comparable plan quality as in DCA plans especially in terms of controlling lower isodose lines. The results of this study suggested that the multi‐target VMAT plans did not necessarily have higher V12Gy per target compared to single‐target DCA plans. On the contrary, as shown in Fig. 1, without proper plan quality management, VMAT plans were prone to have outliers in V12Gy, due to the complexity of VMAT planning. In comparison, the DCA plans showed good consistency because there is less variability in the treatment planning technique. Thus, it is important to have a benchmark data on those important dosimetric metrics in VMAT planning to achieve uniform planning outcome. HyperArc^TM^ technique that was newly developed for Eclipse treatment planning system (Varian Medical Systems, Palo Alto, CA) is an example to standardize the multi‐target VMAT planning process to reduce the variation in treatment planning outcome. While this technique is not available in every treatment planning system or for every treatment unit, the benchmark data on V12Gy (Fig. 1 and Fig. 2) from this study can be used to guide the treatment planning of SIMT cases and can also assist in developing knowledge‐based planning for this type of cases.

The plan quality metrics from VMAT population and DCA population were intercompared in this study for any indications, but not for defining the superiority of one technique over another. In fact, there was no overlap between clinical cases planned with VMAT and planned with DCA. For the cases that have more than 3 targets, VMAT was predominantly used due to consideration of treatment time. The clinical plans in this study included multi‐target cases that had up to 3 targets and were planned with DCA to treat each target separately. Those plans were still referred to as a single‐target plan in this study since each plan had only one target in comparison to multi‐target VMAT plans. It should be noted that the quality metrics of VMAT and DCA plans were not compared on the same datasets in this study. The purpose of this study is not to find out which technique performs better for a given case, but to extract CI and V12Gy from VMAT and DCA plan populations and establish benchmark data on these plan quality metrics for future planning.

Identification of outlier cases from Fig. 1 revealed that a case could have all its targets or a portion of its targets deviating from the average V12Gy level. Re‐optimization of these cases were done by only changing the optimization weight on normal brain sparing. The result of re‐optimization showed a decrease of V12Gy in all outlier targets [Fig. 3(a)]. For the targets whose V12Gy was in the average level in the original plan but went through re‐optimization due to being in the same case with other outlier targets, the V12Gy did not improve much after re‐optimization as expected, but the lower dose spread to normal brain reduced in all cases. By making the 60% isodoseline tighter with the same coverage to target, it is expected that the maximum dose within the target increases.^28^ However, the increased maximum dose in the re‐planned cases was not significant enough to change the plan quality. In most cases where the increase of maximum dose was relatively large, the maximum target dose was still within 115% of the prescription dose [Fig. 3(d)], which was well acceptable in SRS planning. The results of re‐planning showed that it is feasible to use previous planning knowledge to guide VMAT planning and reduce high V12Gy outliers.

Prentou *et al* reported that the CI and gradient index of SIMT VMAT plan deteriorated for an increased number (>6) of simultaneously irradiated targets.^29^ Such deterioration was not observed for V12Gy in our study as shown in Fig. 4. Possible reasons of discrepancy could be different planning systems and delivery systems used in the studies, different isodose lines used for calculation of metrics (gradient index is calculated using 50% isodose volume and V12Gy is calculated from 60% isodose volume), and different planning strategies and priorities used by different institutions, etc. The eight VMAT plans that were identified for re‐optimization in this study included a wide spectrum of number of targets (2, 4, 5, 7, 8, 9, 12, and 15 lesions, respectively), which indicated that there was no direct connection between outlier V12Gy incidence and the number of targets in a single plan. Although V12Gy per target did not show apparent dependence on the number of simultaneously treated targets, we did observe in this study that the low isodose volumes (30% or lower isodose volume) increased with number of targets, which could be optimized further in the future study. In this study, the impact of distance between targets on the V12Gy was also not observed (Fig. 5). A future study can track the planner information for each case to explore potential correlations between the planner experience and the plan quality.

## 5 CONCLUSION

In summary, a benchmark data on SRS planning metrics, CI and V12Gy, has been established to guide future SIMT planning. The multi‐target VMAT plans did not show inferiority dosimetrically when compared to DCA plans. A distribution of normal brain V12Gy as a function of target size was established based on previous clinical cases. The V12Gy for a target in VMAT plan was not impacted by the distance between targets or the total number of targets in the same plan. The results of re‐planning showed that it is feasible to use this knowledge to guide VMAT planning and reduce high V12Gy outliers.

## CONFLICT OF INTEREST

The authors have no conflict of interests to disclose.
